# Root segmentation beyond species boundaries: A generalizable framework for anatomical analysis

**DOI:** 10.1016/j.plaphe.2025.100146

**Published:** 2026-03

**Authors:** Yifei Qian, Yong En Kok, George Janes, Princia Nakombo-Gbassault, Brian Atkinson, Jonathan Atkinson, Molly Hanlon, Darren M. Wells, Michael P. Pound

**Affiliations:** a School of Computer Science, University of Nottingham, United Kingdom; b Centre for Plant Integrative Biology, School of Biosciences, University of Nottingham, United Kingdom; c DIADE, Université de Montpellier, IRD, CIRAD, Montpellier, France; d JEAI AgrobiodiveRCA, Université de Bangui, Bangui, the Central African Republic; e Donald Danforth Plant Science Center, United States of America

**Keywords:** Deep learning, Low-cost, Root anatomical segmentation, Multi-modalities, Limited data

## Abstract

Root anatomical features are critical for plant performance characterization, yet phenotyping at the anatomical scale remains limited by the extreme annotation burden of cellular segmentation. We present a two-stage segmentation framework that greatly reduces annotation requirements while maintaining high accuracy across diverse plant species and imaging conditions. Our approach decomposes multi-class segmentation into species-agnostic tissue identification followed by tissue type classification. By designing robust input representations invariant to imaging artifacts and morphological variations, our framework enables rapid adaptation to new species with fewer than 40 labeled images. Additionally, the first stage automatically generates tissue boundaries, transforming tedious manual tracing into simple tissue labeling. We validate our method on pearl millet, and sorghum root cross-sections from different imaging protocols, achieving state-of-the-art performance while dramatically reducing deployment time. This efficiency breakthrough enables scalable root phenotyping across diverse crop species, accelerating the development of climate-resilient varieties for global food security.

## 1 Introduction

Plant phenotyping plays a key role in basic and applied plant science research, underpinning large-scale genetic discovery and the breeding of more resilient traits [1]. Innovations in this field make a fundamental contribution to the push for global food security, particularly with the combined challenges of global population growth and climate change.

Drought resilience is a particularly crucial crop trait for global food security, especially in arid regions such as sub-Saharan Africa. Root systems play a critical role in plant drought tolerance by controlling water access and transport [2], yet breeding programs have historically overlooked root traits due to the technical challenges of phenotyping underground structures. Recent advances in imaging technologies have made detailed analysis of root anatomy increasingly feasible, revealing that cellular-level features, for example metaxylem diameter and tissue organization, significantly influence plant water use efficiency and drought tolerance [2,3]. For example, a reduction in metaxylem diameter has been shown to improve drought tolerance in wheat by slowing root water uptake and overall plant water use [4].

These findings highlight the critical importance of root anatomical analysis for crop improvement programs. Quantifying these anatomical features requires precise segmentation of cellular structures from high-resolution microscopy images. However, traditional segmentation approaches based on thresholding [5], or region-growing [6], have often focused on architectural traits at a higher scale. Techniques focusing on anatomy [7] have relied on image processing techniques such as watershed segmentation, and typically require extensive user interaction and perform poorly on the complex, variable datasets characteristic of plant tissues.

Recent advances in deep learning, particularly convolutional neural networks (CNNs) and U-Net architectures [8], have revolutionized image segmentation across numerous biological and agricultural domains, offering fully automated and more robust techniques than traditional methods. These approaches have demonstrated significant success in crop monitoring [[9], [10], [11]] and disease detection [12]. Deep learning has also been successfully applied to macroscopic root analysis [[13], [14], [15]], such as root system segmentation from soil images for architectural trait quantification. However, while deep learning tools like PlantSeg [16], Cellpose [17], and Mesmer [18] have established new benchmarks for cellular segmentation performance, they typically rely on large, densely annotated datasets, leaving the challenge of scalable, low-resource segmentation across diverse root tissues largely unresolved.

This difficulty primarily arises from a critical bottleneck: the extreme difficulty of creating annotated datasets. Manual annotation of cellular structures is extremely labor-intensive, requiring expert plant scientists to spend hours precisely delineating complex tissue boundaries and cellular organizations in each image. This annotation burden severely limit the practical application of deep learning approaches.

**Fig. 1:**
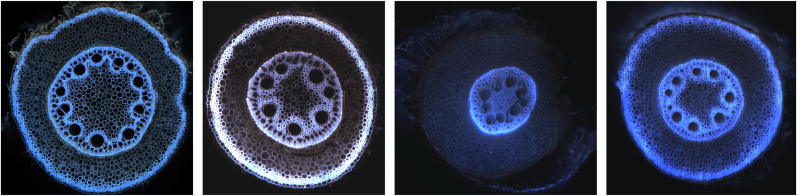
Laser ablation tomography (LAT) images of pearl millet root cross-sections demonstrating imaging variability within the same species due to different acquisition parameters and sample preparation protocols.

The high annotation cost creates two major obstacles for developing robust segmentation models. First, it becomes impractical to collect sufficient training data to handle the significant imaging variability across and within different imaging modalities, such as confocal microscopy, CT and laser ablation tomography (LAT). Different systems, protocol differences and acquisition parameters, sample preparation, and imaging conditions create substantial technical variations that require diverse training examples to address (as shown in Fig. 1). Second, the annotation burden makes it prohibitively expensive to create datasets spanning multiple plant species, yet the dramatic morphological differences between species mean that models trained on one species typically fail when applied to others. These limitations trap researchers in a cycle where each new imaging setup or plant species requires starting the annotation process anew.

The annotation bottleneck is a well-recognized problem in plant imaging, leading to the development of several approaches aimed at reducing the number of labeled samples required for training. These include prototype-based learning [19] for disease segmentation, self-supervised [20] methods combined with edge detection, transfer learning [21] from natural images, and contrastive learning [22] for cross-modality adaptation. While these methods have successfully reduced annotation requirements compared to traditional supervised learning, they still necessitate labeled data from each target domain to achieve acceptable performance. Yet this remaining annotation need poses significant challenges: labeling cellular structures requires extensive expert time, and with thousands of plant species requiring analysis, this creates substantial scalability barriers.

To address these fundamental challenges, we propose a novel two-stage segmentation framework that dramatically reduces annotation requirements while maintaining high performance across diverse imaging modalities, conditions and plant species. Our approach strategically decomposes the complex multi-class segmentation task into two specialized stages: a foreground-background segmentation stage that identifies plant tissue regions, followed by a fine-grained classification stage that distinguishes between different tissue types within the identified regions.

Central to our framework's success is the careful design of robust input representations for each stage, making them invariant to imaging artifacts and species-specific variations. This two-stage architecture, combined with our robust input design, brings two important advantages for scalability. First, when adapting to new plant species, only the second stage requires fine-tuning with minimal data–typically fewer than 40 annotated images–while the first stage generalizes directly. Second, the framework accelerates the annotation process itself: the first stage's output automatically delineates tissue boundaries, allowing experts to focus only on confirming or amending tissue type labels rather than painstakingly tracing cellular structures. Together, these advantages create a multiplicative effect on efficiency, enabling researchers to rapidly deploy our framework across diverse plant species and dramatically accelerating the pace of root phenotyping research for climate-resilient crop development.

In summary, the main contributions of this work are:

• We propose a novel two-stage segmentation framework for root anatomical analysis that decomposes multi-class cellular segmentation into foreground-background segmentation followed by tissue-type classification, significantly reducing the complexity of the learning task and enabling high performance with limited annotations.
• We design robust input representations for cross-domain generalization that make each stage invariant to imaging conditions and species variations, enabling the model to generalize across plant species and different modalities, including LAT, CT and destructive imaging, without extensive retraining.
• We simplify the annotation process by leveraging our first-stage model to automatically generate tissue boundaries, reducing the annotation task from complex cellular delineation to straightforward tissue type labeling.

## 2 Materials and methods

Plant root anatomical segmentation is severely constrained by the labor-intensive and costly process of manual annotations. Our approach introduces a two-stage framework designed to address these limitations systematically. The key insight is that decomposing the complex multi-class segmentation task into hierarchical sub-problems enables both improved generalization and a dramatic reduction in annotation requirements. In this section, we will start with an overview of data sources and preparation. Next, we will present the overall two-stage framework and explain how this decomposition strategy reduces model complexity and annotation requirements. We then detail our robust input preprocessing techniques that enable cross-domain generalization by making the models invariant to imaging artifacts and species-specific variations. Finally, we describe how our framework facilitates rapid annotation workflows, where the first stage output serves as a foundation for efficient expert labeling.

### 2.1 Dataset preparation

LAT uses an ultrafast UV laser to ablate sections from a root sample prior to imaging of the exposed surface, which is illuminated by the UV laser. Moving the sample into the ablation plane allows serial imaging of exposed internal surfaces. This provides a high-resolution and highly detailed image of internal root anatomy. Laser ablation tomography images of roots of pearl millet were produced as detailed in Affortit et al. [23]. Prior to imaging, each root is critically point dried such that no water remains within the root itself. This step is crucial, as residual water would heat during ablation and alter the shape of the cellular structure. The resulting output image is 4096 x 3000 RGB.

A total of 262 images for pearl millet were annotated by domain experts using the semi-automatic Cellset tool [7]. Specifically, the tool generated initial segmentations that were manually corrected and refined by experts to ensure accurate ground truth. We then strategically divided these annotations based on the requirements of our two-stage framework: 177 images were annotated with binary foreground-background masks for training the first stage model, while 85 images received comprehensive multi-class annotations with detailed tissue type labels for training the second stage model. To validate our framework's transferability and annotation efficiency, we additionally collected 40 sorghum root images using LAT. These sorghum images were annotated using our streamlined workflow as detailed in Section 2.4, and used to fine-tune only the second stage model, demonstrating that our framework can adapt to new species with minimal data and annotation effort.

### 2.2 Overall framework

**Fig. 2:**
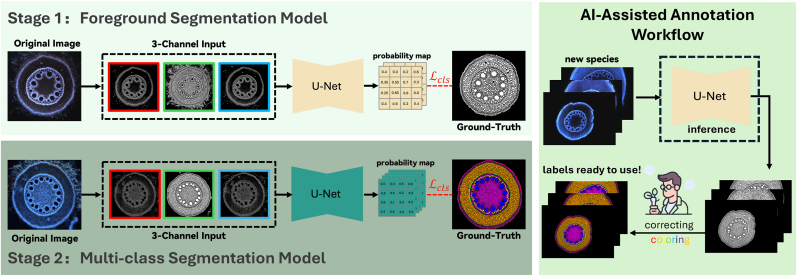
Overview of the proposed two-stage segmentation pipeline. The original LAT image is preprocessed into three distinct channels, which are concatenated as a 3-channel input (analogous to RGB channels) for the U-Net architecture. When adapting to new plant species, the binary segmentation model provides initial tissue masks that can be efficiently refined and labeled by plant scientists, generating training samples for fine-tuning the multi-class model. This design enables rapid deployment across diverse crop species with minimal annotation effort.

Our proposed framework adopts a hierarchical two-stage approach to address the challenges of multi-class root tissue segmentation. As illustrated in Fig. 2, the framework decomposes the complex segmentation task into two specialized models: a binary segmentation model that identifies plant tissue regions (foreground vs. background), followed by a multi-class segmentation model that classifies specific tissue types within the identified regions.

Given the limited availability of annotated data in plant microscopy, we deliberately avoided complex architectural designs that would risk overfitting to small datasets. Deep models with sophisticated modules typically require substantial training data to learn meaningful representations without memorizing training samples. Instead, we employed the standard U-Net [8] architecture for both stages, leveraging its efficient encoder-decoder structure and skip connections to preserve fine-grained spatial information crucial for accurate tissue boundary delineation. The two stages share identical architectures, differing only in the number of output channels to match their respective classification targets. This architectural simplicity, combined with our robust input design, enabled the models to generalize effectively despite the data constraints. We trained both models with a combination of cross entropy and Dice loss, which can be expressed as follows:

(1) $$L_{\text{cls}}=\lambda \cdot L_{\text{CE}}+L_{\text{Dice}},$$

where $L_{\text{CE}}$ is the cross entropy loss:

(2) $$L_{\text{CE}}=-\frac{1}{H\times W}\overset{H}{\underset{i=1}{\sum }}\overset{W}{\underset{j=1}{\sum }}\overset{C}{\underset{c=1}{\sum }}y_{ij}^{c}\log\left({\hat{y}}_{ij}^{c}\right),$$

and $L_{\text{Dice}}$ is the dice loss:

(3) $$L_{\text{Dice}}=1-\frac{2}{C}\overset{C}{\underset{c=1}{\sum }}\frac{\sum_{i=1}^{H}\sum_{j=1}^{W}y_{ij}^{c}{\hat{y}}_{ij}^{c}}{\sum_{i=1}^{H}\sum_{j=1}^{W}y_{ij}^{c}+\sum_{i=1}^{H}\sum_{j=1}^{W}{\hat{y}}_{ij}^{c}},$$

where *H* × *W* is the image size, *C* is the number of classes, $y_{ij}^{c}$ and ${\hat{y}}_{ij}^{c}$ are the ground truth and predicted probability for class *c* at pixel (*i*, *j*), respectively, and *λ* is a balancing factor.

The key advantage of this two-stage design is its superior cross-species generalization. While a single-stage multi-class model fails to transfer between species due to morphological variations, our binary model learns robust features to distinguish plant tissue from background that generalize across different crops. When adapting to new species, this pre-trained binary model can be directly applied, and only the second stage requires fine-tuning with minimal labeled data. Moreover, in our AI-Assisted Annotation Workflow, the binary model's output could be leveraged to provide initial tissue boundaries, enabling plant scientists to focus on tissue type labeling rather than manual boundary delineation, dramatically accelerating the annotation process.

### 2.3 Robust input preprocessing

The key to robust cross-domain generalization lies in designing input representations that capture essential tissue structures while remaining invariant to imaging variations. Our preprocessing strategy for the binary segmentation model is motivated by a consistent observation in LAT images: cell walls exhibit higher intensity than intracellular regions across different imaging protocols and plant species.

To capture this structural information in a robust manner, we applied adaptive thresholding to create a binary representation of tissue organization. Specifically, we employed Gaussian blur with a 15 × 15 kernel to reduce noise while preserving edge information, followed by adaptive thresholding using a Gaussian-weighted mean with inverse binary output. For each pixel (*i*, *j*), the binary output is determined as:

(4) $$B\left(i,j\right)=\left\{\begin{array}{ll} 255\; & ifI^{\prime }\left(i,j\right)<\mu_{\mathrm{local}}\left(i,j\right)+C,\\ 0\; & otherwise, \end{array}\right)$$

where *I*′(*i*, *j*) is the pixel value after Gaussian blur, *μ*_*local*_(*i*, *j*) is the Gaussian-weighted mean within a 35 × 35 neighborhood, and *C* is a constant offset which is set to 1.

While the adaptive threshold output provides a clear binary representation, using it alone as neural network input may result in loss of fine-grained structural information. To address this, we augmented the input with gradient orientation features extracted at multiple scales, as orientation captures the geometric structure of tissue boundaries while remaining invariant to local contrast variations – a key requirement for cross-domain generalization. For each scale with kernel size *k* ∈ {3, 7}, we computed:

(5) $$g_{x}^{k}\left(i,j\right)=\left(I*S_{x}^{k}\right)\left(i,j\right),\;g_{y}^{k}\left(i,j\right)=\left(I*S_{y}^{k}\right)\left(i,j\right),$$

where $S_{i}^{k}$ and $S_{j}^{k}$ are Sobel kernels of size *k* × *k*. The gradient orientation and magnitude were then calculated as:

(6) $$\theta^{k}\left(i,j\right)=arctan2\left(g_{y}^{k}\left(i,j\right),g_{x}^{k}\left(i,j\right)\right),$$

(7) $$m^{k}\left(i,j\right)=\sqrt{g_{x}^{k}{\left(i,j\right)}^{2}+g_{y}^{k}{\left(i,j\right)}^{2}}.$$

To focus on significant edges while suppressing noise, we applied magnitude-based filtering:

(8) $$\theta_{f}^{k}\left(i,j\right)=\left\{\begin{array}{ll} \frac{\theta^{k}\left(i,j\right)+\pi }{2\pi }\times 255\; & \text{if }m^{k}\left(i,j\right)>m_{75}\\ 0\; & \text{otherwise} \end{array}\right)$$

where *m*_75_ is the 75^th^ percentile of gradient magnitudes across the image. By combining binary tissue masks (*B*_adp._) with multi-scale gradient orientations ($\theta_{f}^{3}$ and $\theta_{f}^{7}$), this preprocessing strategy enables the binary model to learn robust representations that generalize across species and imaging protocols.

For the second stage, tissue type classification, we constructed a 3-channel input tensor by concatenating binary segmentation output from the first stage, denoted as *B*_out_ with the gradient features ($\theta_{f}^{3}$ and $\theta_{f}^{7}$) computed above. Our design is motivated by two key observations. First, using raw RGB images directly for multi-class segmentation suffers from high variability across imaging conditions, making models brittle and species-specific. Second, we observed that plant scientists can reliably annotate tissue types using only binary masks, suggesting that tissue identity is largely encoded in spatial patterns rather than color or intensity values. The binary mask provides robust tissue localization independent of imaging variations, while the gradient features preserve structural information that might be lost in binary conversion. This combination enables the model to focus on the geometric and organizational patterns that define each tissue type, allowing our model to be rapidly fine-tuned for new plant species while maintaining high accuracy.

### 2.4 Efficient expert labeling

Pixel-by-pixel annotation of images is highly resource-intensive, particularly for high-resolution images such as LAT. When labeling from scratch, researchers must invest substantial time and effort in boundary delineation and detail refinement. Moreover, precise boundary annotation often requires domain expertise to distinguish target regions from the background, making the process susceptible to subjective bias and annotation fatigue when handling large image datasets.

However, by leveraging the pre-trained binary segmentation model, the annotation process can be significantly accelerated. The model automatically generates initial binary masks for unlabeled images, requiring researchers to only perform minor corrections and assign tissue type labels. Researchers may also strategically select representative samples for annotation based on the model's predictions, for example by focusing on challenging cases, rather than annotating all images indiscriminately. This active selection strategy ensures that annotation effort is directed toward the most informative samples, maximizing the improvement in model performance per annotated image. In our experiments, we manually selected samples with high cell classification error rates as candidates for correction.

This AI-assisted workflow not only enhances annotation efficiency but also ensures consistency in annotation quality. The resulting annotated images can then be used to fine-tune the species-specific multi-class model, enabling rapid deployment for phenotypic analysis of new plant species.

## 3 Results

### 3.1 Datasets

In this study, we primarily utilized a pearl millet dataset containing 262 LAT images, as detailed in Section 2.1. For training the stage 1 binary segmentation model, we used 177 images with only binary foreground-background masks as the training set, while reserving the remaining 85 images with complete multi-class annotations for evaluation. The multi-class annotations comprise 9 distinct classes: background, cortex, metaxylem, stele tissue, aerenchyma, vascular bundle, sclerenchyma, epidermis and endodermis. The separate subset of 85 images used for Stage 2 was further split into 55 for training and 30 for testing. These 30 testing images were kept entirely separate and were strictly excluded from the training sets of both the Stage 1 and Stage 2 models.

Additionally, we collected 40 sorghum root images and applied our AI-assisted annotation workflow to generate multi-class labels, using 25 for training and 15 held out for testing the stage 2 model.

### 3.2 Implementation details

**Experimental Settings:** Both stages of our framework employed identical U-Net architectures and training configurations with ImageNet-pretrained ResNet-34 [24] as a backbone, differing only in the number of output channels (2 for binary segmentation, 9 for multi-class). We implemented our models using PyTorch and trained them on a single NVIDIA RTX A6000 GPU.

For both stages, the models were optimized using the Adam optimizer with a learning rate of 10^−4^, and a weight decay of 10^−3^. The balancing weight (*λ*) was set to 0.1. We trained the models for 500 epochs with a batch size of 8, with the final model selected for evaluation on the test set. We set the *λ* = 0.1 for all experiments. Data augmentation was applied during training to improve generalization, including random horizontal flips, random resizing to 768 × 768, and random cropping of 768 × 768 patches. For evaluation on test set, images were resized to 1024 × 1024 for full-image prediction.

**Evaluation Metrics:** The performance of the segmentation model was measured by using the following metrics:

**Mean Intersection over Union (mIoU)** computes the average IoU across all classes:

(9) $${\text{IoU}}_{c}=\frac{\left|P_{c}\cap G_{c}\right|}{\left|P_{c}\cup G_{c}\right|}=\frac{TP_{c}}{TP_{c}+FP_{c}+FN_{c}}.$$

(10) $$\text{mIoU}=\frac{1}{C}\overset{C}{\underset{c=1}{\sum }}{\text{IoU}}_{c}$$

**Dice Coefficient** measures the overlap between prediction and ground truth:

(11) $${\text{Dice}}_{c}=\frac{2|P_{c}\cap G_{c}|}{\left|P_{c}|+|G_{c}\right|}=\frac{2\times TP_{c}}{2\times TP_{c}+FP_{c}+FN_{c}},$$

(12) $$\text{Dice}=\frac{1}{C}\overset{C}{\underset{c=1}{\sum }}{\text{Dice}}_{c}$$

where *P*_*c*_ and *G*_*c*_ represent the predicted and ground truth regions for class *c*, *TP*_*c*_, *FP*_*c*_, and *FN*_*c*_ are true positives, false positives, and false negatives for class *c*, respectively. For multi-class segmentation, we report the mean values across all classes (including background).

### 3.3 Results and ablation study

In this section, we systematically evaluate our two-stage framework across multiple dimensions to demonstrate its key advantages.

#### 3.3.1 Overall performance

We first compared our approach with a single-stage baseline that performs direct multi-class segmentation from RGB images. This baseline model used the same U-Net architecture but took raw RGB images as input without any preprocessing, and was trained end-to-end without our preprocessing pipeline. To comprehensively evaluate both within-species performance and cross-species generalization capabilities, we conducted experiments on both pearl millet and sorghum datasets. For each species, we trained both methods on the respective training set and evaluated on both the corresponding test set and the complete dataset of the other species. We present the results in Table 1 and Table 2.

**Table 1:** Performance comparison between the proposed two-stage framework and the direct single-stage multi-class segmentation baseline when trained on Pearl millet dataset.

	Pearl millet (Test)		Sorghum (Full)	
	Dice	mIoU	Dice	mIoU
Baseline (Multi-class)	**0.7734**	**0.6407**	0.5545	0.4144
Ours	0.7510	0.6123	**0.6357**	**0.4926**

**Table 2:** Performance comparison between the proposed two-stage framework and the direct single-stage multi-class segmentation baseline when trained on sorghum dataset.

	Sorghum (Test)		Pearl millet (Full)	
	Dice	mIoU	Dice	mIoU
Baseline (Multi-class)	0.6327	0.5116	0.2555	0.1668
Ours	**0.6408**	**0.5180**	**0.4192**	**0.2879**

Based on the results, we observe distinct performance patterns that highlight the advantages of our proposed framework. In within-species evaluation, the performance differences between the two methods were marginal: the baseline achieved slightly higher scores on the larger pearl millet dataset with approximately 2.0 % improvement in both metrics, while our method performed marginally better on the smaller sorghum dataset. This pattern suggests that our approach may be particularly advantageous when training data is limited, as the sorghum training set contains approximately half the samples of pearl millet. Our method's deliberate removal of redundant information while preserving essential anatomical features appears to provide better data efficiency in low-resource scenarios. Importantly, these marginal differences should be interpreted with caution, as the ground-truth annotations are inherently imperfect due to the manual labeling process. Given the high-resolution nature of our images, achieving perfect boundary delineation is extremely challenging, and small numerical improvements may simply reflect overfitting to annotation inconsistencies rather than genuine performance gains.

However, the marginal within-species performance differences become insignificant when evaluating cross-species generalization capabilities. The removal of redundant, species-specific information enabled our framework to achieve remarkably superior cross-species performance. When the model trained on pearl millet was directly applied to sorghum data, our method demonstrated substantial improvements over the baseline, with gains exceeding 8 % in both Dice and mIoU metrics. This pattern was consistently observed in the reverse scenario: models trained on sorghum and tested on pearl millet showed even more dramatic improvements, with our method achieving 16.4 % higher in Dice score.

These results have important implications for practical plant phenotyping applications. The superior cross-species generalization capability suggests that models trained on one plant species could potentially be applied to other species, requiring only selective annotation and sampling of poorly performing samples in the new species rather than comprehensive dataset labeling. This dramatically reduces annotation requirements, making our framework particularly valuable for agricultural research where annotated datasets are often limited and expensive to obtain.

**Fig. 3:**
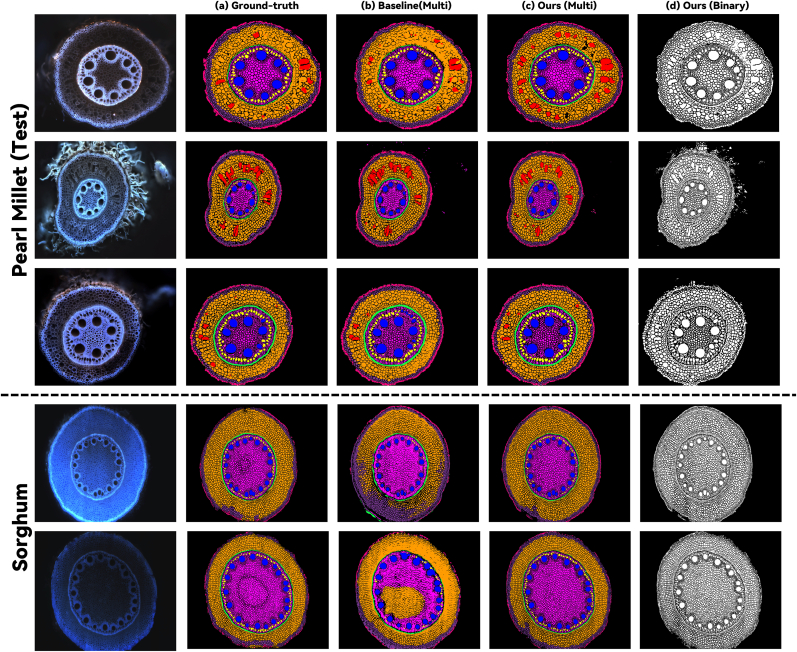
Visualizations of the performance of the foreground and multi-class segmentation models trained on pearl millet.

We also provide qualitative results from models trained on pearl millet in Fig. 3. As illustrated in the examples, our method, benefiting from the prior knowledge embedded in the binary segmentation stage, typically achieved superior performance in delineating cellular boundaries. Additionally, our model demonstrated strong cross-species generalizability, as evidenced by the consistent segmentation quality when applied across different plant species.

#### 3.3.2 Ablation study on the effectiveness of robust input

To validate the effectiveness of our robust input preprocessing, we conducted ablation studies on both stages of our framework. We systematically evaluated the contribution of each input channel by comparing: (1) our complete three-channel input design, (2–4) models using each channel individually, and (5) a baseline using raw RGB images directly. This evaluation was performed for both the binary segmentation model in Stage 1, which uses adaptive threshold and two gradient orientations, and the multi-class segmentation model in Stage 2, which uses binary mask and two gradient orientations. All experiments were performed on the pearl millet dataset.

As shown in Table 3, the results demonstrate that our multi-channel design significantly outperformed single-channel alternatives in both stages. For binary segmentation, our complete three-channel approach achieved the highest performance on both metrics, outperforming the binary baseline by 1.53 % and 2.40 % on Dice and mIoU respectively. For Stage 2, the gradient orientation channels and *B*_out_ achieved comparable performance on the Dice metric. Both stages demonstrated that the three channels provided complementary information that compensates for the information loss inherent in our input preprocssing strategy, where substantial visual details are deliberately removed to focus on essential anatomical features. This validates our design philosophy of using multiple input modalities to preserve critical structural information while filtering out redundant visual content.

**Table 3:** Ablation study on the effectiveness of robust input.

Stage	$\theta_{f}^{3}$	$\theta_{f}^{7}$	*B*_adp._	*B*_out_	Baseline (RGB)	Dice	mIoU
1	*✓*					0.8441	0.7393
		*✓*				0.8374	0.7277
			*✓*			0.8503	0.7597
					*✓*	0.8698	0.7750
	*✓*	*✓*	*✓*			**0.8851**	**0.7990**
2	*✓*					0.7312	0.5886
		*✓*				0.7169	0.5713
				*✓*		0.7228	0.5809
					*✓*	**0.7734**	**0.6407**
	*✓*	*✓*		*✓*		0.7510	0.6123

#### 3.3.3 Phenotypic trait verification

To validate the biological utility of our framework, we conducted a downstream phenotypic analysis comparing manual measurements against those derived automatically from our multi-class model's segmentation output. We utilized a holdout set of 25 test images each for both pearl millet and sorghum. We compared automated measurements of Root Diameter, Stele Diameter, and Cortex Width against manual ground-truth values obtained using Fiji. Manual measurements were taken by measuring each root via 4 intersecting lines at 0°, 45°, 90°, 135°. These measurements were averaged to produce a final value for each phenotypic trait.

**Fig. 4:**
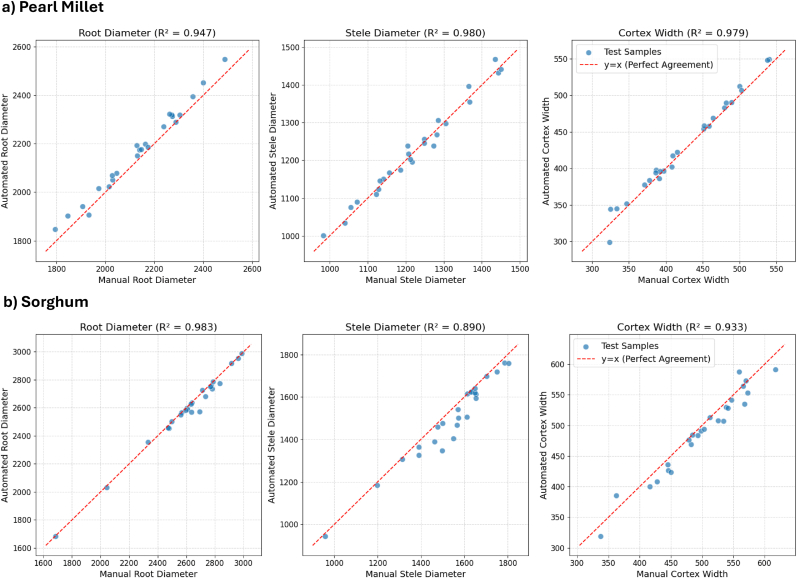
Correlation between ground truth manual measurements and automated measurements derived from multi-class segmentation model's output.

As illustrated in Fig. 4, our framework demonstrates high agreement (*R*^2^ > 0.89) with manual measurements for both pearl millet and sorghum datasets. These results confirm that the segmentation output captures essential anatomical features with sufficient precision to replace manual measurement in high-throughput workflows.

## 4 Discussion

The recent development of high-throughput phenotyping approaches, such as LAT, has made possible the profiling of thousands of root samples necessary for the genetic dissection of anatomical traits of interest [25], creating a workflow bottleneck in quantifying anatomical traits from image data. The need for automated software approaches is thus pressing, yet faces a second workflow bottleneck: the domain-expert annotation of ground truth images.

In this work, we present a two-stage segmentation framework that fundamentally addresses the cross-species domain adaptation challenge in root anatomical phenotyping. By effectively managing domain shifts, our approach simultaneously enables the rapid analysis of large datasets and provides a simplified annotation pipeline for generating ground truths in new species. Our approach decomposes the complex multi-class segmentation task into species-agnostic tissue detection followed by tissue-type classification, achieving robust performance across different plant species while dramatically reducing annotation requirements. On top of that, our downstream analysis confirms that this segmentation accuracy translates directly into precise phenotypic trait quantification, validating the practical utility of the framework for high-throughput biological research. The experimental results on pearl millet and sorghum demonstrate that our framework maintains competitive within-species accuracy while exhibiting superior cross-species generalization – a critical capability for scaling root phenotyping across diverse crop varieties.

The motivation for our hierarchical design stems from a fundamental scalability challenge in root phenotyping: morphological and anatomical variations across plant species render models trained on one species ineffective on others, yet the prohibitive cost of manual annotation makes it impractical to create comprehensive datasets for every species of agricultural importance. This domain shift problem has confined most existing approaches to single-species applications, severely limiting their impact on crop improvement programs that must evaluate diverse germplasm. Our framework directly addresses this bottleneck by explicitly separating species-invariant features, such as tissue presence and boundaries, from species-specific patterns of tissue type organization.

**Fig. 5:**
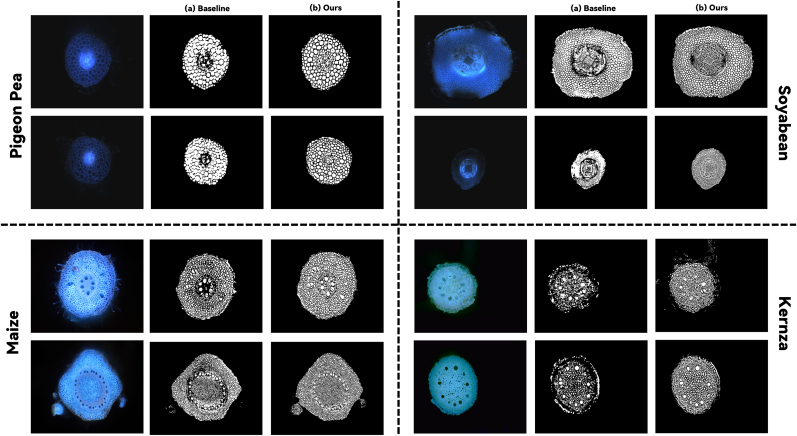
Performance comparison between our binary segmentation model and baseline on four unseen plant species.

The robustness of our binary segmentation model arises from its focus on fundamental anatomical principles shared across plant species. By leveraging the consistent observation that cell walls exhibit higher intensity than intracellular regions regardless of species or imaging protocol, our preprocessing pipeline extracts features that remain valid across domains. In Fig. 5, we demonstrate the performance of our binary segmentation model across four different plant species. Notably, our binary model was trained exclusively on pearl millet data, yet the results show that our approach achieves superior segmentation performance on these previously unseen species compared to the RGB-based baseline model. This species-agnostic capability of the binary stage significantly reduces annotation burden for new species deployment. Since the binary model can immediately provide accurate tissue boundaries for any new species, researchers need only perform simple region labeling and minor boundary corrections rather than tedious pixel-level tracing, transforming what traditionally required hours of expert annotation into a task achievable in minutes.

Furthermore, the preprocessing strategy for our second-stage model deliberately removes species-specific visual information while preserving structural patterns that encode tissue identity. By using binary masks and gradient orientations rather than raw intensity values, the model learns geometric and organizational features that transcend imaging conditions and species boundaries. This design philosophy explains why our framework achieves superior cross-species generalization compared to traditional RGB-based approaches that must learn to navigate both species-specific visual variations and anatomical classification simultaneously. Moreover, this species-invariant feature extraction suggests that performance could be further enhanced by incorporating data from multiple species during training, as diverse anatomical patterns would enrich the model's understanding of fundamental tissue organization.

To validate this hypothesis, we conducted joint training experiments using combined pearl millet and sorghum data and the results are presented in Table 4. These findings demonstrate the advantages of our species-invariant approach: while the RGB-based baseline experienced performance degradation on pearl millet despite improvements on sorghum, our preprocessing-based framework maintained stable performance on pearl millet while achieving improvements on sorghum. This contrast highlights that traditional RGB methods suffer from interference between species-specific visual characteristics, whereas our approach enables additional species data to enhance performance without negative interference on existing species.

**Table 4:** Performance comparison between the proposed two-stage framework and the direct single-stage multi-class segmentation baseline when trained on joint dataset.

	Pearl millet		Sorghum	
	Dice	mIoU	Dice	mIoU
Baseline (Multi-class)	0.7489	0.6123	0.7073	0.5661
Ours	**0.7512**	**0.6125**	**0.7311**	**0.5921**

**Fig. 6:**
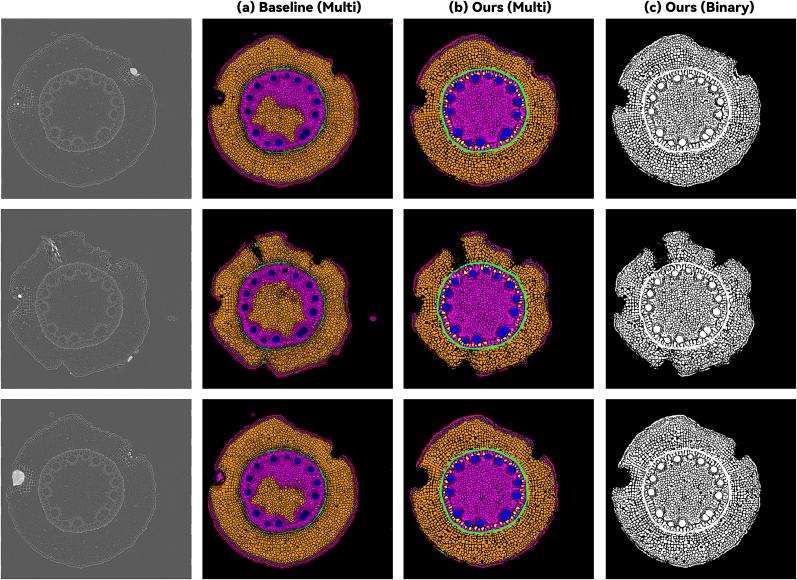
Cross-modal generalization: segmentation results when applying models trained on LAT images to CT scan data.

Most significantly, our approach exhibits inherent cross-modal generalization capability owing to its geometry-based learning paradigm rather than modality-specific visual features. To demonstrate this capability, we applied the model trained on the above joint LAT dataset directly to sorghum root x-ray CT scans without any retraining or fine-tuning. As shown in Fig. 6, our method exhibits excellent performance on the CT modality, while the RGB-based baseline model produces poor segmentation results with artifacts throughout cellular regions and fails to distinguish between different tissue types, requiring substantial re-annotation and fine-tuning for deployment.

In conclusion, this framework opens new possibilities for large-scale biological imaging applications where robust cross-domain and cross-modal generalization is essential. Future work could extend this approach to other plant organs and explore its applicability to broader biological imaging domains where morphological consistency exists beneath surface-level variations.

## Author contributions

Y. Qian: conceptualization, software, methodology, and writing—original draft. Y.Kok: software, writing—review and editing All reviewed and edited the manuscript. G. Janes: data collection and annotation. P. Nakombo-Gbassaul: data collection. J. Atkinson: writing—review and editing. M. Hanlon: data collection. D. Wells and M.Pound: project conception, funding, supervision, and writing—review and editing. All reviewed and edited the manuscript.

## Funding

This work was supported by the Biotechnology and Biological Sciences Research Council (grant number BB/Y513908/1), the Agence National de la Recherche (SorDrought grant ANR-23-CE20-0052, and the New Roots for Restoration Biology Integration Institute (NSF 2120153).

## Data availability

The dataset and models are available at https://doi.org/10.5281/zenodo.17726414 and https://doi.org/10.5281/zenodo.17737703, respectively. The source code is hosted at https://github.com/janetkok/Root-Segmentation-Beyond-Species-Boundaries.

## Declaration of competing interest

The authors declare the following financial interests/personal relationships which may be considered as potential competing interests: Michael Pound reports financial support was provided by Biotechnology and Biological Sciences Research Council. If there are other authors, they declare that they have no known competing financial interests or personal relationships that could have appeared to influence the work reported in this paper.
